# Rapid recombinant protein expression in cell-free extracts from human blood

**DOI:** 10.1038/s41598-018-27846-8

**Published:** 2018-06-22

**Authors:** David Burgenson, Chandrasekhar Gurramkonda, Manohar Pilli, Xudong Ge, Abhay Andar, Yordan Kostov, Leah Tolosa, Govind Rao

**Affiliations:** Center for Advanced Sensor Technology (CAST) and Department of Chemical Biochemical and Environmental Engineering (CBEE), University of Maryland Baltimore County, Baltimore, Maryland USA

## Abstract

Several groups have recently reported on the utility of cell-free expression systems to make therapeutic proteins, most of them employing CHO or *E. coli* cell-free extracts. Here, we propose an alternative that uses human blood derived leukocyte cell extracts for the expression of recombinant proteins. We demonstrate expression of nano luciferase (Nluc), Granulocyte-colony stimulating factor (G-CSF) and Erythropoietin (EPO) in cell-free leukocyte extracts within two hours. Human blood is readily available from donors and blood banks and leukocyte rich fractions are easy to obtain. The method described here demonstrates the ability to rapidly express recombinant proteins from human cell extracts that could provide the research community with a facile technology to make their target protein. Eventually, we envision that any recombinant protein can be produced from patient-supplied leukocytes, which can then be injected back into the patient. This approach could lead to an alternative model for personalized medicines and vaccines.

## Introduction

Cell-free expression of proteins is finding rapid application as an alternative to recombinant cells for producing research grade proteins. Several commercial kits are available. In contrast to research grade material, production of biologically-derived medicines or therapeutics involves large, complex and expensive cell culture bioreactors^1^. The high cost of production is due to the maintenance of the expression host, rigorous protein expression and purification, and packaging and transport through a cold chain to keep the product stable until it reaches the patient^2^. Cell-free technologies for producing biologics have not yet been shown to be economical and also have not navigated regulatory requirements for safety, which mandate rigorous validation and toxicity studies. Approaches to miniaturize bioreactors to shift production to the point-of-care have been explored^3^. However, these rely on the use of living cells and do not solve the problem of host maintenance and lengthy production times. As an alternative, the use of cell-free extracts for the production of recombinant proteins on demand has been recently reported^4,5^. The cell extracts contain a majority of the cellular machinery capable of producing functionally active proteins. Cell-free expression reduces production time from weeks to hours and lyophilization of the extracts allow for protein production at the point of need. Recently, the cell-free system from CHO cells^6^, insect^7^ and *E. coli*^8^ have been used to produce therapeutic targets^4,5^. The use of rabbit reticulocyte derived cell free systems to produce potential therapeutic proteins was described several decades ago^9^. In this paper, we introduce the concept of generating the recombinant proteins in leukocyte extracts from human blood. Human blood may be an alternative to existing cell-free protein expression systems currently available in the market and may potentially involve fewer regulatory hurdles for use in humans.

## Results and Discussions

**Figure 1:**
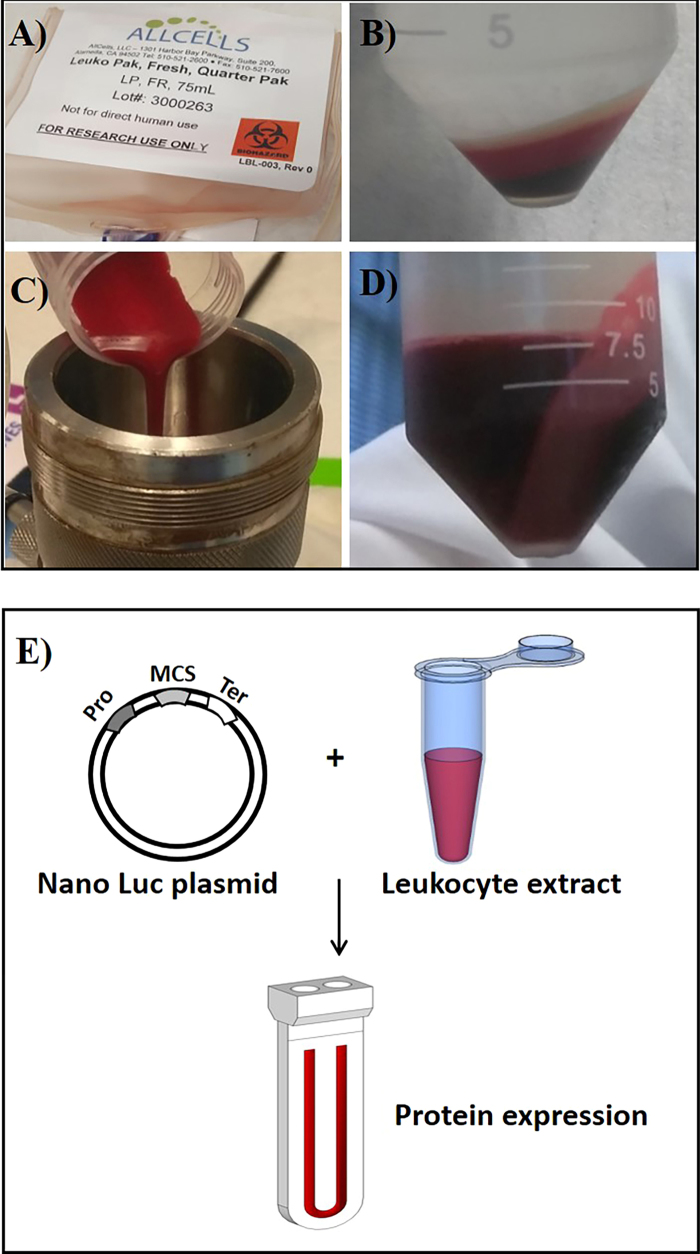
Production of human leukocyte extract and Nluc expression. (**A**) Leukopak was purchased form the AllCells, Inc. (**B**) The cells were clarified by centrifugation followed by washing with wash buffer (see materials and methods section). A white leukocyte layer is seen in the top layer of the centrifuged sample. (**C**) Washed cells were reconstituted in extraction buffer and lysed using nitrogen cavitation method (**D**) Leukocyte extract collected from the vessel was further clarified and the final extract was collected in Eppendorf tubes and stored at −80°C. (**E**) Expression of Nano-luciferase in leukocytes extracts - the Leukocyte cell-free protein synthesis system consists of leukocyte cell lysate and solutions for reaction mix and dialysis buffer. *In vitro* transcription and translation reactions (IVT) were setup in micro dialysis device for overnight expression (~16 h) and samples were collected and tested for luciferase activity.

**Figure 2:**
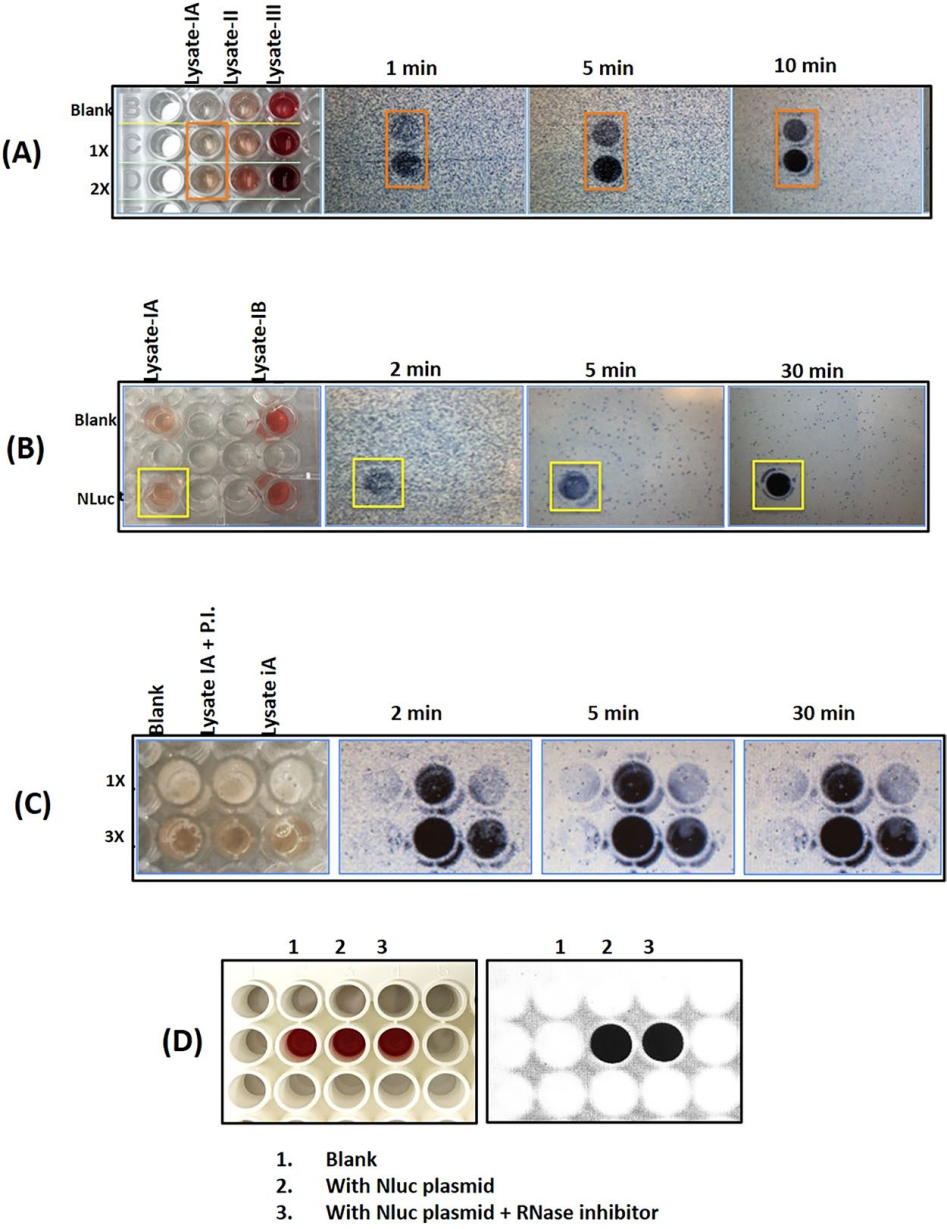
(**A**) Spiking experiments showed that luciferase luminescence is a reliable detection method for protein expression in blood, since hemoglobin interference proved problematic with colorimetric detection. Luminescence signal was detected in an ECL imager (Thermo Fisher Scientific, Rockford, IL, USA) for different lengths of time with auto-gain. We tested different lysing methods. Lysate-IA represents the supernatant fraction obtained after lysis with nitrogen cavitation method. Lysate 1B was the corresponding pellet re-suspended in extraction buffer. Lysate-II was obtained by lysing the cells with hypotonic solution containing PBS. Lysate-III was obtained by shearing the cell suspension by passing through an 18-gauge needle. Only blood lysate-IA showed luminescence with an increasing dose response. (**B**) Lysates made from the same source but using two different methods were tested along with blanks (no DNA). All samples received substrate followed by imaging. Nluc signal was detected in Lysate-IA (supernatant of nitrogen cavitation method) but not in either blanks or Lysate-IB (the pellet of nitrogen cavitation method was resuspended in extraction buffer and tested for Nluc expression). (**C**) Protease inhibitors (P.I.) were tested to see if expression would improve and was found to increase the Nluc activity about 2 to 3 fold when compared with no P.I. 1 ×, 2 × and 3 × represents Nluc substrate concentration. (**D**) Reproducibility of Nluc expression in leukocyte cell-free system. Nluc was expressed with and without RNase inhibitor. Luciferase analysis using Nluc substrate analyzed on ECL imager.

The *in vitro*-coupled transcription and translation system was carried out in a small-scale dialysis device consisting of 100 µl of the reaction mixture in 1.3 mL dialysis buffer separated by 10 kDa dialysis membrane. As a proof of principle, we demonstrated the expression of Luciferase (Nluc), Granulocyte-colony stimulating factor (G-CSF), and Erythropoietin (EPO) using a leukocyte cell-free extract. The results provide evidence that the leukocyte cell-free system is able to produce the recombinant proteins in a few hours. We prepared the cell-free extract by lysing the leukocytes using different methods, namely, Dounce homogenizer (data not shown), hypotonic lysis (lysate-II), and passing through an 18-gauge needle (lysate-III). Lysis by nitrogen cavitation method (lysate-IA) is shown in Fig. 1A–D. For the optimization studies of the lysing conditions, Nluc, a deep-sea shrimp luciferase, was used as test protein^10^. Nluc luminescence is stable and not affected by the components of blood extract, making it an ideal reporter for assessing the extract potency. Protein synthesis of Nluc with leukocyte lysate under different lysing conditions was performed over a period of 16 h (Fig. 1E). The results (Fig. 2A) show that only the lysate prepared by nitrogen cavitation (lysate IA) produces Nluc and was chosen as the method for further study. Resuspension of the pellet (lysate IB) did not result in Nluc expression suggesting that the active components are present in the supernatant, lysate IA (Fig. 2B). The addition of protease inhibitor further enhanced the expression of Nluc, but RNase inhibitor did not have a significant effect (Fig. 2C,D).

**Figure 3:**
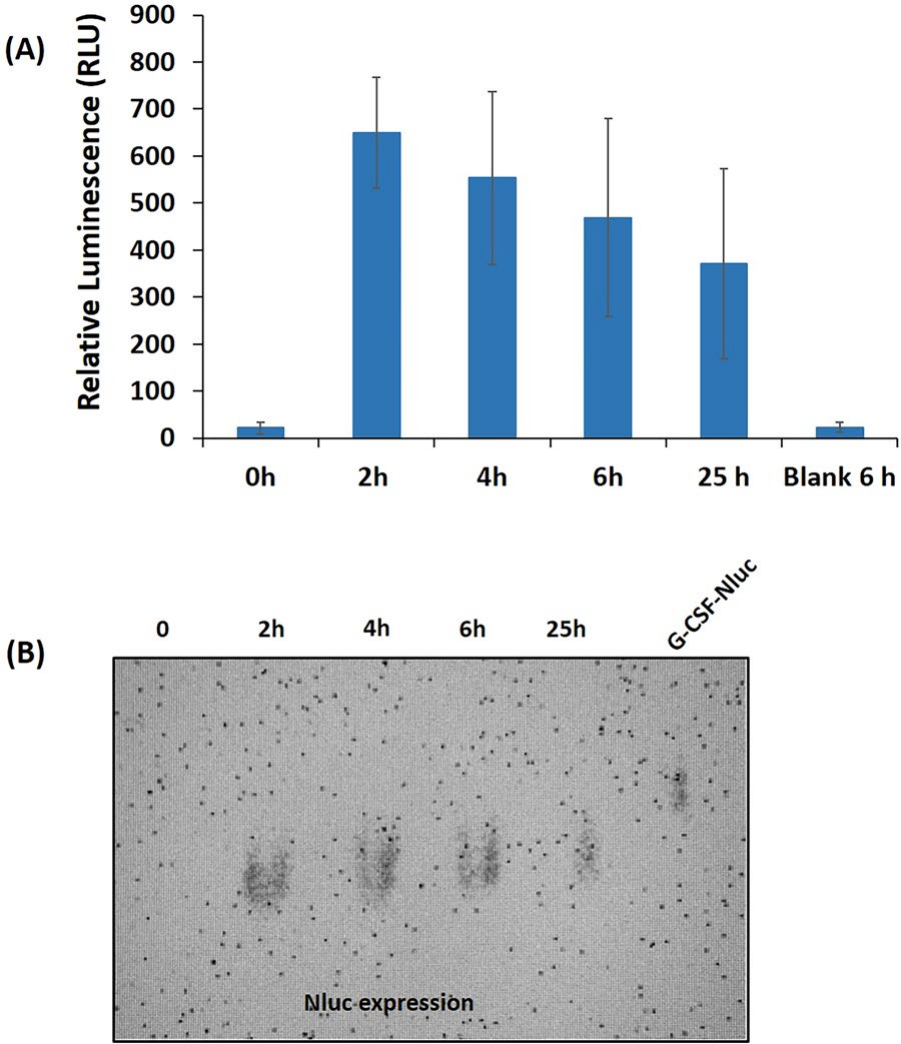
(**A**) Time-course expression and kinetic analysis of luciferase expression using leukocyte cell-free system. The error bars represent 90% confidence intervals (n = 3). (**B**) Native-PAGE showing the expression of luciferase and G-CSF-Nluc.

In Fig. 3A, the expression of Nluc showed proteolytic degradation over time, with maximum protein expression at 650 RLU in 2 h of production time. The native PAGE analysis confirms the same (Fig. 3B), suggesting that a 2 h expression time is ideal. Note that this short expression time is not possible in cell-based protein expression. Apart from Nluc, we have also expressed G-CSF tagged with Nluc and expressed for 25 h. The G-CSF-Nluc was expected to run at a higher molecular weight compared with Nluc, which was confirmed by the shift on the native PAGE despite expression without protease inhibitors (Fig. 3B).

**Figure 4:**
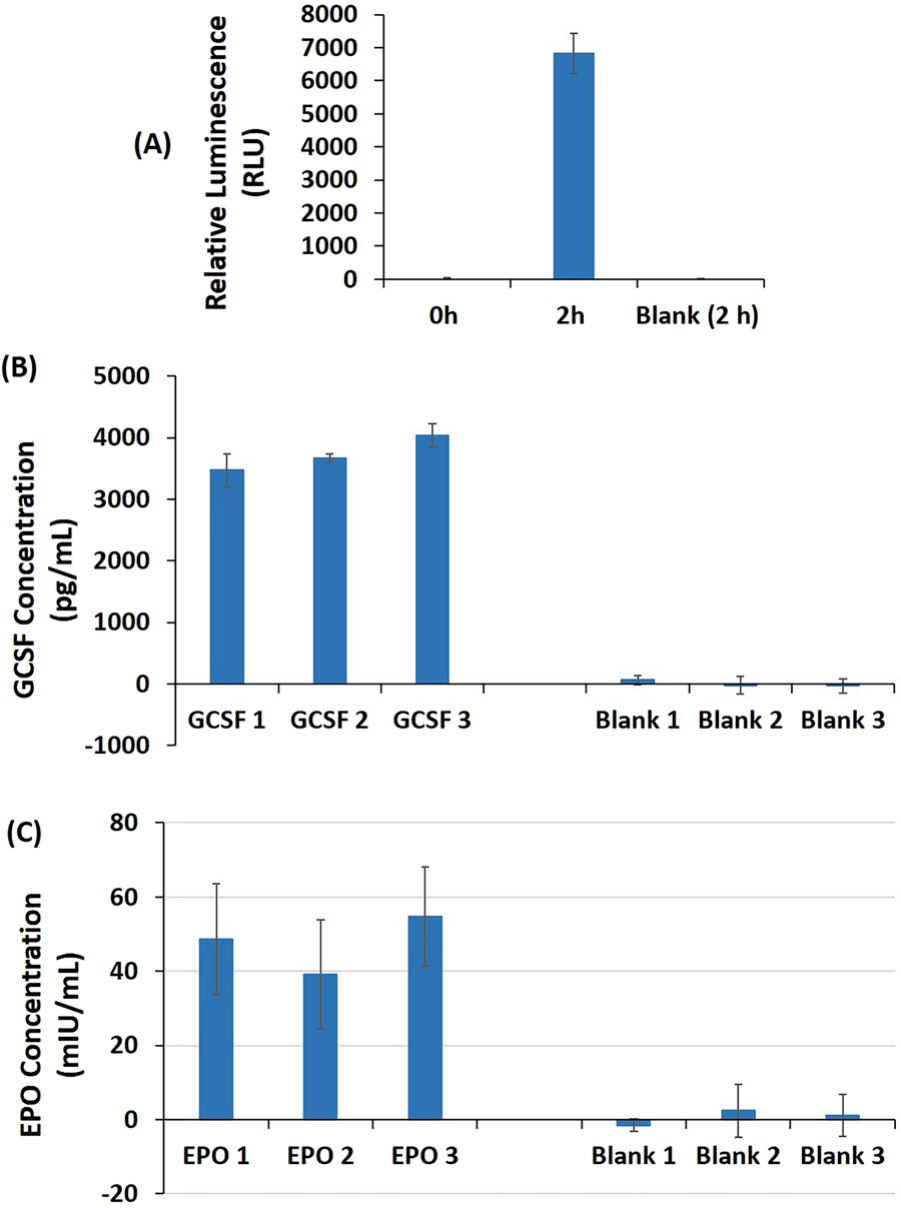
(**A**) Expression of luciferase using leukocyte cell-free system. (**B**) Quantification of G-CSF produced (expression time −2 h) using leukocyte cell-free system. G-CSF standard curve generated using a quantitative Sandwich ELISA (G-CSF Human ELISA, Abcam, USA) following the manufacturer’s instructions. All materials required for the analysis was provided in the kit. For the standard curve, a dilution series containing 0 to 500 pg/mL of G-CSF standard was prepared. (**C**) Quantification of EPO (expression time − 2 h) produced using leukocyte cell-free system. For reference standard protein concentrations, a dilution series containing 0 to 100 mIU/mL of EPO standard was prepared. The concentration of EPO in the harvest sample was determined using a quantitative Human EPO ELISA Kit, Abcam, USA) following the manufacturer’s instructions. All materials required for the analysis were provided in the kit. Blank represents no-DNA control. The error bars represent 90% confidence intervals (n = 3).

To further evaluate the performance of leukocyte cell-free system, we chose a set of model proteins including G-CSF (used to minimize the effects of radiation therapy), and EPO (used to increase red blood cell count in anemic patients). These model proteins were expressed for 2 h in the leukocyte coupled *in vitro* transcription and translation system. The results demonstrate the production of Nluc, G-CSF and EPO in the blood cell extracts (Fig. 4). The data confirm that the leukocyte cell-free system can produce the therapeutic proteins of interest. Using this system, we were able to produce nanogram level quantities per mL reaction (Table 1). Further codon optimization is underway, including replacement of the T7 RNA polymerase with human RNA polymerase to make the cell-free reaction bioequivalent. Relatively high reproducibility of the results in the expression of G-CSF, EPO, and Nluc (Fig. 4) show potential for therapeutic protein production. However, we do see yield variations based on the donor and these will need to be further studied. Alternatively, one can imagine lyophilized leukocyte extracts sourced from pooled donors as a relatively inexpensive source of human cell extracts for producing reagent grade proteins on demand, as has been demonstrated with lyophilized CHO extracts^4^. Further demonstration of efficacy of therapeutic proteins or vaccines derived from the leukocyte extracts will be determined pending optimization of product yields and concomitant purification of the protein as needed. At this point, this work serves as a proof-of-principle, since expression yields are too low for product characterization by tools such as mass spectrometry.

**Table1:** Leukocytes origin and preparation of extracts^1^.

Donor information	Purchased date	
	May 11, 2017	October 10, 2017
Blood Type	O+	B+
Age	21	24
Sex	Male	Male
BMI	25.4	26.7
Tobacco Use	No	No
Race	Non-Hispanic White	White
Medication Use	No	No
Viral Status^2^	Negative	Negative
Total Cell Count^3^	0.3 × 10^10^ Cells	0.5 × 10^10^ Cells
Preparation of leukocyte extract		
Total Protein concentration (after lysis)^4^	20.4 µg/µL	47.1 µg/µL
Expression time (h) and model proteins tested and validated	2 h (Nluc) 4 h (Nluc) 6 h (Nluc) 25 h (Nluc and G-CSF-Nluc) 16 h (Nluc and G-CSF)	2 h (Nluc, G-CSF, EPO)
Reactivity	Reporter assay (Nluc) ELISA (G-CSF)	Reporter assay (Nluc) ELISA (G-CSF and EPO)
Yields	~650 RLU (Nluc) ~2.0 ng/ml (G-CSF by ELISA)	~6800 RLU (Nluc) ~3.7 ng/ml (G-CSF by ELISA) ~47 mIU/ml (EPO by ELISA)

^1^Leukopak was purchased from AllCells, USA.

^2^Tested for HIV, HBV, HCV, and CMV.

^3^Total cell count includes all the cell types present in the blood.

^4^Protein estimation was analyzed using 660 protein assay from Thermo Fischer Scientific, Rockford, IL, USA.

Further studies are in progress to determine other factors necessary for higher expression levels. In particular, in the absence of nuclease treatment, the rate of protein synthesis slows over time (results not shown). This may be due to the presence of other mRNAs competing with the production of the protein of interest.

Efforts to address the drawbacks in the development of personalized medicine are in the pipeline^10^. It is possible that one approach may be to use the patient’s own blood as a source for manufacturing biologics at the point-of-care once the system’s yields are improved. In addition, technologies to express proteins from cell-free systems at the point-of-care are also emerging. We have recently described a completely automated system that can produce purified biologics in a few hours^11^.

## Methods

### Plasmids

The rDNA encoding the recombinant proteins Nano-Luciferase His, G-CSF-His, G-CSF Nano-Luciferase His, and, EPO-His (Gurramkonda *et al*., 2018) were sub-cloned into the IVT expression vector, pT7CFE1-CHis using a combination of *NdeI, XhoI*, and *BglII* and restriction sites. *NdeI* and *XhoI* were used for G-CSF and EPO inserts, *XhoI* and *BglII* were used for Nano-Luciferase. Similarly, rDNA for a truncated version of human GADD34, an accessory protein to the IVT reaction, was sub-cloned into the pT7CFE1-CMyc vector using *NdeI* and *XhoI* restriction sites. Plasmids were transformed into ZYMO DH5a *E. coli* cells. These cells were allowed to proliferate overnight. The next day, plasmid DNA were isolated using Zymo-Giga plasmid isolation kit following the manufacturer’s guidelines. GADD34 is co-expressed with the protein of interest.

### Preparation of extracts

Leukocytes (60 mL pack) were purchased from (AllCells, CA, USA). The cells were collected in a 4 × 50 mL centrifuge tubes (15 mL of cells/tube). To the 15 mL leukocytes, 20 mL of wash buffer [35 mM Hepes-KOH pH 7.5 + 135 mM NaCl + 11 mM glucose] was added and mixed gently. This suspension was centrifuged at 3000 rpm for 5 min at 4 °C, and washed three times with a buffer (35 mM Hepes–KOH, pH 7.5, 135 mM NaCl, and 11 mM glucose) and once with an extraction buffer (35 mM Hepes–KOH, pH 7.5, 135 mM KAc, 30 mM KCl, and 1.65 mM MgAc). The cell pellet was then resuspended in extraction buffer (Example: 1 g cell pellet*0.8 = 0.8 mL of extraction buffer) and was disrupted by nitrogen pressure (300 psi) in the cell disruption chamber. Cell homogenates were centrifuged twice at 10,000 g for 5 min at 4 °C, and the supernatant (4.5 mL) was stored in 500 µL aliquots at −80 °C. The protein concentration was determined using 660 protein assay from Thermo Fischer Scientific (Rockford, IL).

### Preparation of *In vitro* translation system

The Leukocyte cell-free protein synthesis system is comprised of leukocyte cell lysate and solutions for the Reaction Mix and Dialysis Buffer^4,5,11^. All components were allowed to come to room temperature. The components were then added in the following order: 50 µL lysate, 20 µL 5 × reaction mix, 0.4 µg GADD34 plasmid, and 4 µg cDNA plasmid. The mixture is brought to a total volume of 100 µL with nuclease-free water.

### Expression in Microdialysis devices

Reactions were carried out following manufacturer’s recommendations with minor modifications for CHO cell free expression in continuous exchange cell-free (CECF) protein expression format. The IVT reaction was injected into a 0.1 mL size, 10 kDa MWCO Pierce^®^ 96-well Microdialysis device (Thermo Fisher Scientific, Rockford, IL) using a pipette. Loaded devices were then immersed in 2 mL Eppendorf tube containing 1.3 mL of 1X dialysis buffer. The tubes were then incubated in a pre-warmed incubator at 30 °C (Thermomixer C, Eppendorf). Reactions were carried out for their respective timeframe at 30 °C with constant shaking at 600 rpm.

### Nluc Native PAGE analysis

A 5 µL aliquot of Nluc containing harvest was mixed with 10 µL Native sample buffer and run at 100 V for 30 minutes under native conditions using a 4–15% SDS PAGE gel. The gel was then removed and Nluc substrate diluted 1:100 in PBS pH 7.4, was added on top of the gel and imaged using a chemiluminescent imager (Thermo-fisher scientific, myECL) with a matte black background.

### Quantitative analysis of G-CSF and EPO by ELISA

The concentration of G-CSF in the cell harvests was determined using a quantitative Sandwich ELISA (G-CSF Human ELISA, Abcam, USA) following the manufacturer’s instructions. All materials required for the analysis was provided in the kit. For the standard, a dilution series containing 0 to 500 pg/mL of G-CSF standard was prepared. The clarified samples were diluted accordingly with a buffer containing 0.1% BSA in PBS (pH = 7.2) and analyzed in triplicate. Briefly, 100 µL of each standard and sample was added to appropriate wells and incubated for 2.5 hours at room temperature. The wells were washed with wash buffer and added with 100 µL of biotinylated G-CSF antibody and further incubated for 1 hour at room temperature. After washing with wash buffer, 100 µL of the HRP-streptavidin solution was added to each well and incubated for 45 minutes at room temperature. Then, 100 µL of TMB substrate solution was added followed by incubation for 30 minutes, and the reaction subsequently halted by adding 50 µL of stop solution. Finally, absorbance at 450 nm was measured using a SpectraMax^®^ M5 Multi-mode microplate reader (Molecular Devices, Sunnyvale, CA).

The concentration of EPO in the Leukocyte lysate harvests was determined using a quantitative ELISA (Erythropoietin (EPO) Human ELISA Kit, Abcam, USA) following the manufacturer’s instructions. All materials required for the analysis were provided in the kit. For reference standard protein concentrations, a dilution series containing 0 to 100 mIU/mL of EPO standard was prepared. EPO expressed Leukocyte Lysate standards were diluted 1 to 5 using sample diluent provided with the kit. The 96 well plate provided with the kit is pre-coated with primary antibody to EPO. The plate was washed twice with 400 µL of 1x wash buffer before any samples were added. A 50 µL of sample diluent was added to sample (non-standard) wells. 100 µL of each standard was added to standard wells, and 50 µL sample (final dilution of 1 in 10) were sample wells. 50 µL of 1x Biotin Conjugated Antibody was then added to all wells, and the plate was incubated at 25 °C and 400 rpm. The wells were washed with 400 µL of 1x wash buffer 6 times. 100 µL of Streptavidin-HRP and further incubated for 15 minutes at 25 °C and 400 rpm. The wells were then again washed with 400 µL of 1x wash buffer 6 times. Then, 100 µL of TMB substrate solution was added followed by incubation for 10 minutes at 25 °C covered to avoid direct exposure to light. The reaction was subsequently halted by adding 100 µL of stop solution, briefly mixed by tapping the side of the plate, and absorbance read at 450 nm immediately using a SpectraMax^®^ M5 Multi-mode microplate reader (Molecular Devices, Sunnyvale, CA).
